# Antagonism of nicotinic acetycholinergic receptors by CN‐105, an apoE‐mimetic peptide reduces stroke‐induced excitotoxicity

**DOI:** 10.1002/ctm2.677

**Published:** 2022-01-24

**Authors:** Miaomiao Xue, Shuya Li, Mingzhi Xu, Li Yan, Daniel T. Laskowitz, Brad J. Kolls, Gang Chen, Xiaohong Qian, Yongjun Wang, Haifeng Song, Yi Wang

**Affiliations:** ^1^ State Key Laboratory of Proteomics Beijing Proteome Research Center National Center for Protein Sciences (Beijing) Beijing Institute of Lifeomics Beijing China; ^2^ Department of Neurology Beijing Tiantan Hospital Capital Medical University Beijing China; ^3^ China National Clinical Research Center for Neurological Diseases Beijing China; ^4^ ICE Bioscience Inc. Beijing China; ^5^ Duke Clinical Research Institute Duke University School of Medicine Durham North Carolina USA; ^6^ Department of Neurology Duke University Durham North Carolina USA; ^7^ Department of Anesthesiology Duke University Durham North Carolina USA; ^8^ Aegis‐CN, LLC Durham North Carolina USA; ^9^ Guangdong Cerebtron Biotech Ltd. Guangdong China

Dear Editor:

This letter describes our work identifying the neuronal targets of a clinical‐stage stroke therapeutics CN‐105, and proposing a novel neuroprotective strategy involving nAChR antagonism. Stroke is a devastating disease with high morbidity and mortality. CN‐105 was originally designed to mimic the anti‐inflammatory activities of endogenous apolipoprotein E (apoE). Despite its proven efficacy in various animal models of brain injury and well‐established safety profile in clinical trials,^1^, ^2^ our understanding of CN‐105’s mechanism of action remains incomplete. Early reports suggested that apoE‐derived peptides and a number of oligoarginine species may interact directly with various neuronal targets including the nicotinic acetylcholine receptors (nAChR).^3^, ^4^ The long‐held view regarding nAChR was that its activation was neuroprotective, best exemplified by the cognitive‐enhancing effects of nAChR agonists or positive allosteric modulators.^5^ One common role of nAChR, which received limited attention in stroke, is its potentiation of glutamate release at the presynaptic terminal.^6^ Glutamatergic neurotransmission is arguably the primary reason for the propagation of excitotoxicity in stroke.^7^, ^8^ Although postsynaptic nAChR activated by ACh or nicotine could desensitize proximal NMDA receptors,^9^ we hypothesized that acute action of nAChR antagonist on presynaptic neurons may serve to downregulate the detrimental cascade associated with glutamate excitotoxicity.

After given supratherapeutic dosing of CN‐105 several orders of magnitude higher than clinical practice, Cynologous monkey exhibited symptoms of mydriasis and ptosis. A gradual recovery to normal activity took about two serum clearance half‐lives of CN‐105 (4 h, Table S1; Figure S1). In C57BL6 mice, CN‐105 had an LD_80_ of 25 mg/kg (Figure 1A) with symptoms of spasm and respiratory suppression. Following intubation and mechanical ventilation, dosing of 50 mg/kg was not associated with mortality (Figure 1A), but led to extended sedation after the removal of isoflurane anaesthesia (Movie S1).

**FIGURE 1:**
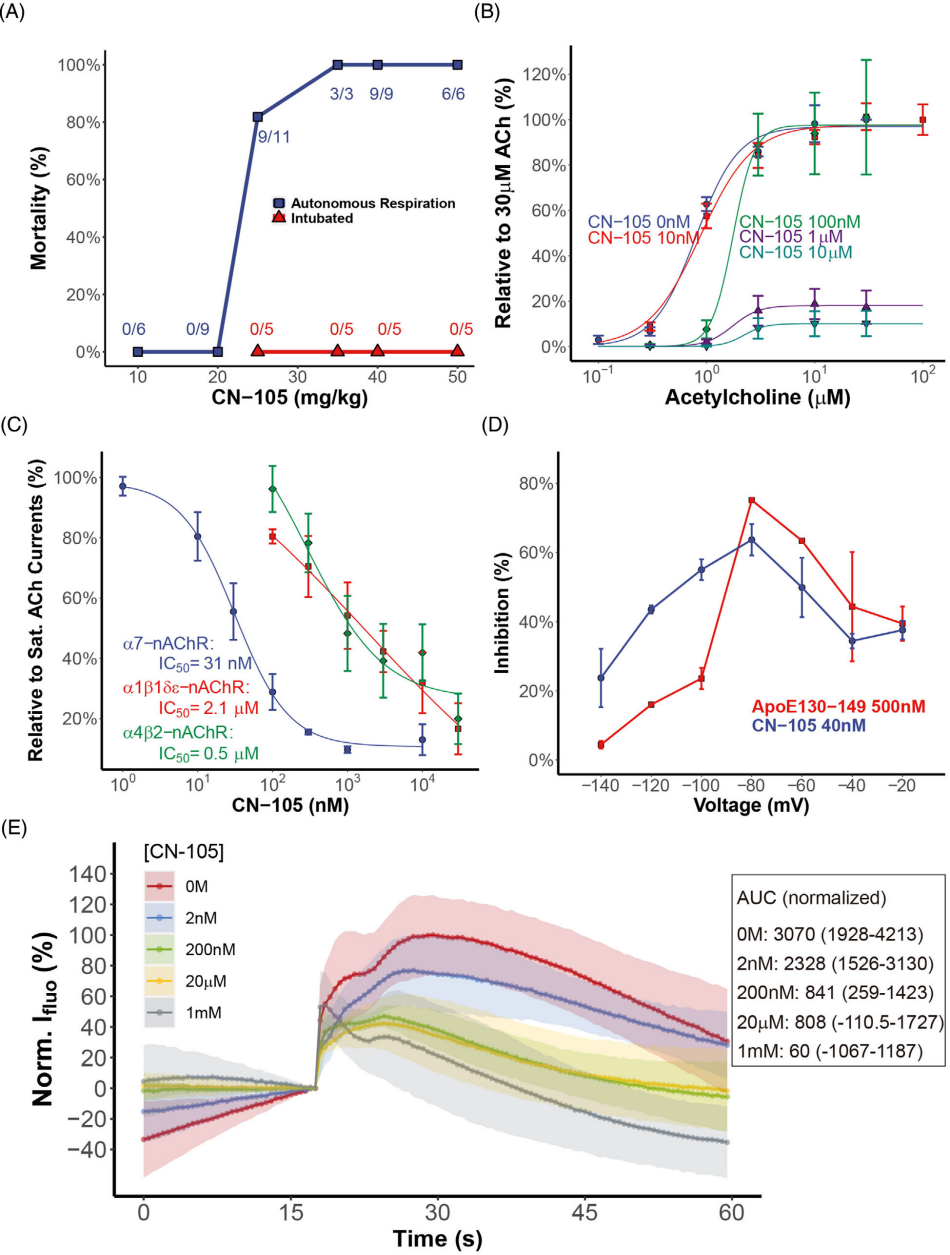
(A) Mortality rates of C57BL6 mice after a single tail vein injection of CN‐105. Red triangles corresponded to mice under mechanical ventilation with isoflurane anaesthesia. The numbers of mice died from respiratory suppression over the total numbers of the test mice were marked beside each datum. (B) Dose–response curves of ACh stimulation of α7‐nAChR in the presence of various concentrations of CN‐105. All current data were normalized to the current induced by saturating concentration of ACh (30 μM). The data were fitted to a three‐parameter logistic model in R with the drc package. The EC_50_ for ACh with CN‐105 concentration from 0 to 10 μM were: 0.80 ± 0.05 μM, 0.88 ± 0.07 μM, 1.76 ± 0.35 μM, 1.74 ± 0.50 μM, 2.23 ± 1.06 μM, with *p*‐values being <0.001, <0.001, <0.001, 0.003 and 0.51, respectively. (C) Dose–response curves of CN‐105's inhibition of α7‐, α1β1δε‐ and α4β2‐nAChR. The data were fitted to a four‐parameter logistic model in R with the drc package. The *p*‐values of data fitting for the three subtypes were <0.001, 0.43 and <0.001, respectively. (D) Voltage dependencies of α7‐nAChR inhibition by CN‐105 (40 nM) or ApoE130‐149 (500 nM), both at concentrations close to their respective IC_50_. (E) Calcium influx imaging with the Ca^2+^ sensitive dye AM4 on rat primary neuron stimulated by 1 mM ACh. CN‐105 at various concentrations was added to cell culture prior to the imaging experiment. For a better comparison between groups, fluorescence intensities were normalized to the maximum mean fluorescence intensity of the 0 M CN‐105 group. All plots were shifted so that the fluorescence intensity immediately prior to ACh injection was zero. Each curve was the mean of five replicates with the 95% confidence interval depicted in the same colour

Given apoE‐peptides’ antagonistic activity upon nAChR,^3^, ^10^ we hypothesized that the respiratory suppression of CN‐105 was due to its inhibition of nAChRs present in various respiratory control pathways. An initial electrophysiological study using an HEK293 cell line overexpressing α7‐nAChR showed that 10 μM CN‐105 strongly suppressed the ACh induced current, similar to the inhibitory effect of apoE (Figure S2). In the presence of 10 nM to 10 μM CN‐105, we observed a typical antagonistic suppression of the ACh‐induced currents (Figure 1B). Such effect was sub‐type specific, as the IC_50_ of CN‐105 differed by two orders of magnitude for α7‐, α4β2‐ and α1β1δε‐nAChR (Figure 1C), the latter of which might be responsible for CN‐105’s respiratory toxicity at supratherapeutic dosage. The voltage dependency of CN‐105 was measured in parallel with apoE130‐149 (Figure 1D), in which we observed maximal inhibition at –80 mV. In the primary culture of rat neurons, 1 mM ACh triggered strong calcium influx, which was significantly diminished by a priori incubation with CN‐105 prior to ACh infusion (Figure 1E). These results indicated that CN‐105 may block the ACh‐induced influx of Ca^2+^ and the propagation of action potential (AP).

Based on the reported binding site of the apoE140‐148 peptide on nAChR,^10^ we constructed a model of the α7 nAChR‐CN‐105 complex. The putative binding site of CN‐105 was proximal to the orthosteric binding site for ACh (Figure S3A) and would most likely block the binding of ACh. Unsurprisingly, the arginine side chains of CN‐105 were involved in extensive hydrogen bonds (Figure S3B). Poisson–Boltzmann analyses of the complex structure (Figure 2A) showed that positive charges of CN‐105 significantly weakened the negative electric field that is critical for the passage of cations, diminishing the propensity of cation inflow. The two preceding neutral residues were vital for the nAChR activity (Figure S3B). The peptide in which the first valine was replaced by an arginine lost most of its nAChR activity (Table S2) in spite of possessing an extra positive charge.

**FIGURE 2:**
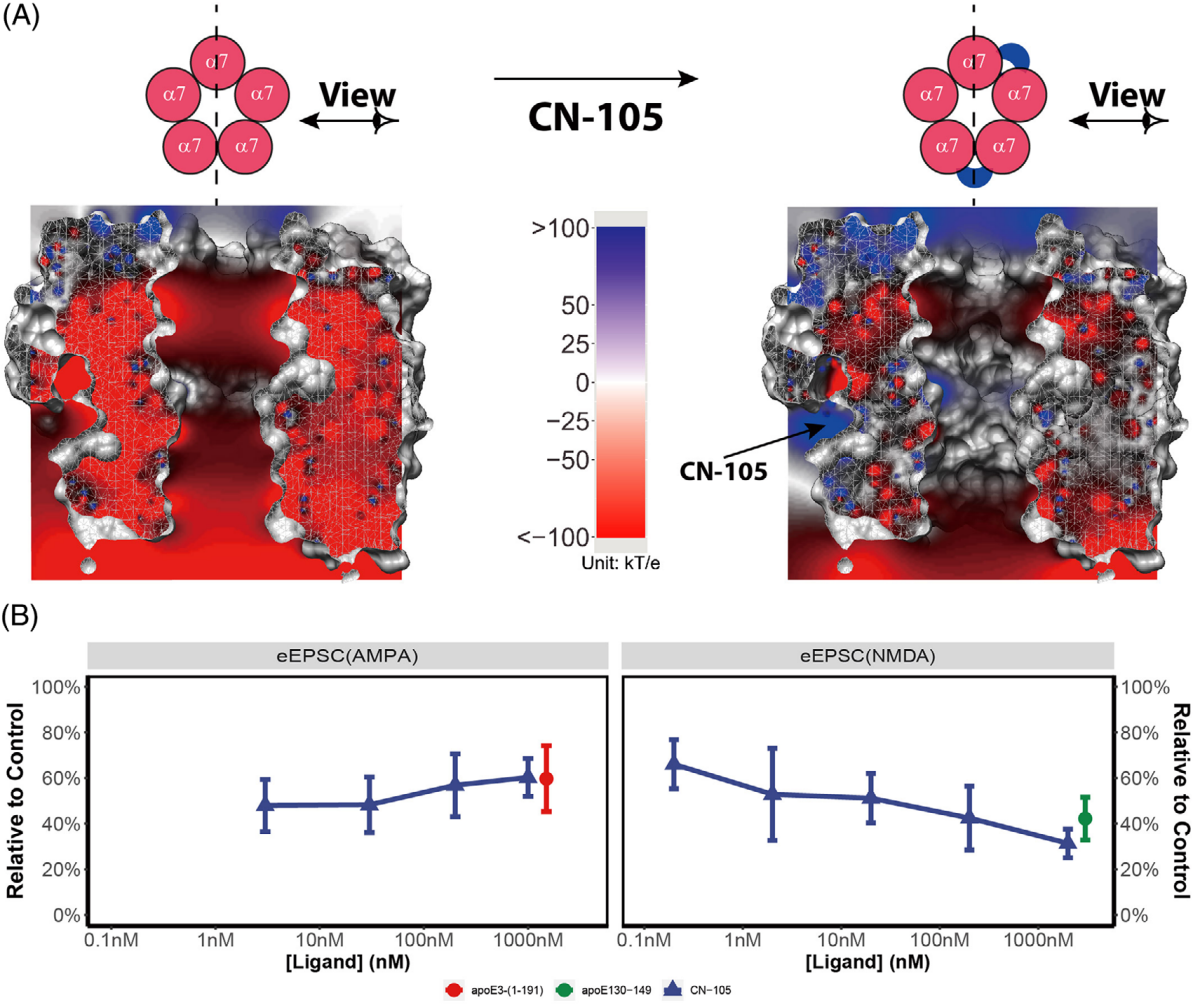
(A) Electrostatic potential map of the ECD portion of the α7‐nAChR before and after the binding of CN‐105. The channel structure was depicted in surface mode and clipped in the Y‐Z plane (dashed line) for a better view of the channel. Graphics were generated in Chimera. (B) Normalized evoked excitatory postsynaptic current (EPSC) signals of AMPA receptor and NMDA receptor, recorded in the presence of CN‐105, apoE130‐149 (3 μM) or apoE3‐(1‐191) (1 μM). Each datum on the plot was the mean of EPSC data from four replicates, except that the 200 nM CN‐105 data for eEPSC(AMPA) and eEPSC(NMDA) were averages of eight repeats, respectively (Figure S4)

We next studied whether CN‐105’s inhibition of nAChR and nAChR‐mediated calcium influx would affect downstream release of glutamate. In rat hippocampal brain slices, evoked excitatory postsynaptic current (eEPSC) mediated by NMDA receptor and AMPA receptor were significantly lower in the presence of CN‐105 than the control levels (Figure 2B, Figure S4). CN‐105 did not directly inhibit glutamate‐induced currents in NMDA or AMPA receptors (Figure S5). Rather, CN‐105’s attenuation of the spontaneous EPSC (sEPSC) frequency and the reversion by the cholinesterase inhibitor donepezil (Figure 3A), suggested an intercellular and most likely presynaptic action of CN‐105. Confining the agonistic effect of donepezil to nAChR by adding the muscarinic AChR inhibitor atropine exhibited a similar effect to adding donepezil alone, indicative of an nAChR‐dependent mechanism. When tetrodotoxin was added to block all AP firings, CN‐105 was no longer able to affect the NMDA receptor‐mediated EPSC (Figure 3B). These results suggested that CN‐105 could suppress the presynaptic release of glutamate in an AP‐ and AChR‐dependent manner. That is, nAChR^+^ glutamatergic neurons may be the primary cellular target of CN‐105 in suppressing excitatory neurotransmission in the brain.

**FIGURE 3:**
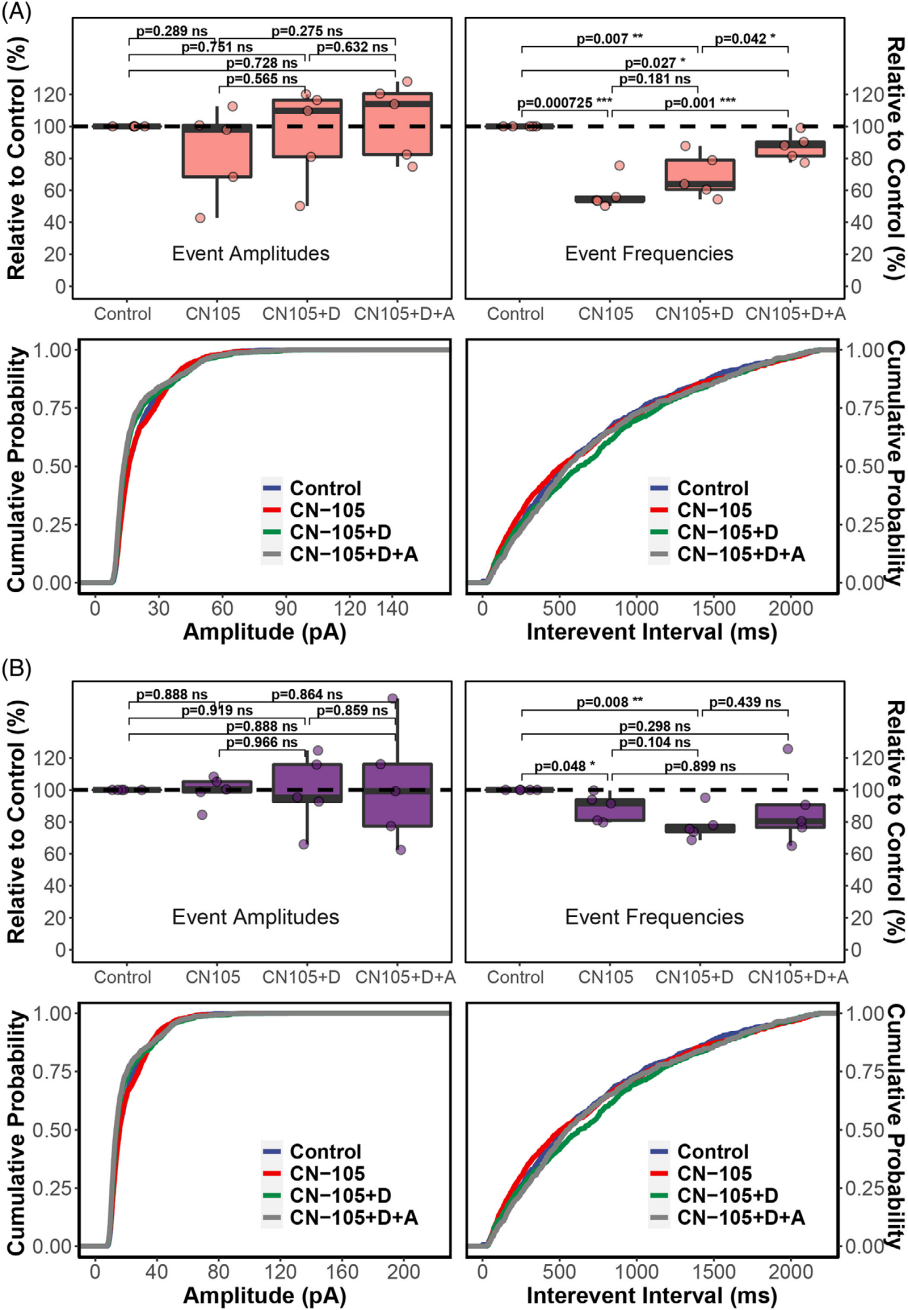
(A) Excitatory postsynaptic current (EPSC) amplitudes and event frequencies of rat hippocampal brain slice recorded in the spontaneous mode (sEPSC). CN‐105, donepezil and atropine concentrations were 200, 20 and 1 μM, respectively. (B) EPSC amplitudes and event frequencies of rat hippocampal brain slice recorded in the presence of TTX (mEPSC). CN‐105, donepezil (labelled as “D”) and atropine (labelled as “A”) were 200, 20 and 1 μM, respectively. Statistics (*t*‐test) and empirical cumulative distribution functions (ECDF) were calculated using the “rstatix” and “ggpubr” packages in R

In a rat model of transient ischemic stroke, CN‐105 (0.1 to 0.4 mg/kg) significantly reduced the infarct volume, similar to the group given the free‐radical scavenger edaravone (Figure 4A). At the infarct margin of the primary somatosensory cortex, we enumerated the α7‐nAChR^+^ cells which had higher density in the CN‐105 treated groups than in the vehicle‐treated groups (Figure 4B). The number of β3‐GABA_A_R^+^ cells in this area was unaffected by CN‐105 (Figure 4C). Similar to the trend of α7‐nAChR^+^ cells, the overall density of viable neurons (as NeuN^+^ cells) was higher in CN‐105 treated groups (Figure 4D), which suggested that specific interactions between CN‐105 and neurons carrying α7‐nAChRs in the somatosensory cortex may help protect neuronal tissues from the ischemic‐reperfusion injury. Our current understanding of CN‐105’s neuronal mechanism of action is depicted in Figure 4E. Although chronic inhibition of the cholinergic pathway may be detrimental, our results demonstrated that acute and selective antagonism of nAChR may actually protect the brain from excitotoxicity.

**FIGURE 4:**
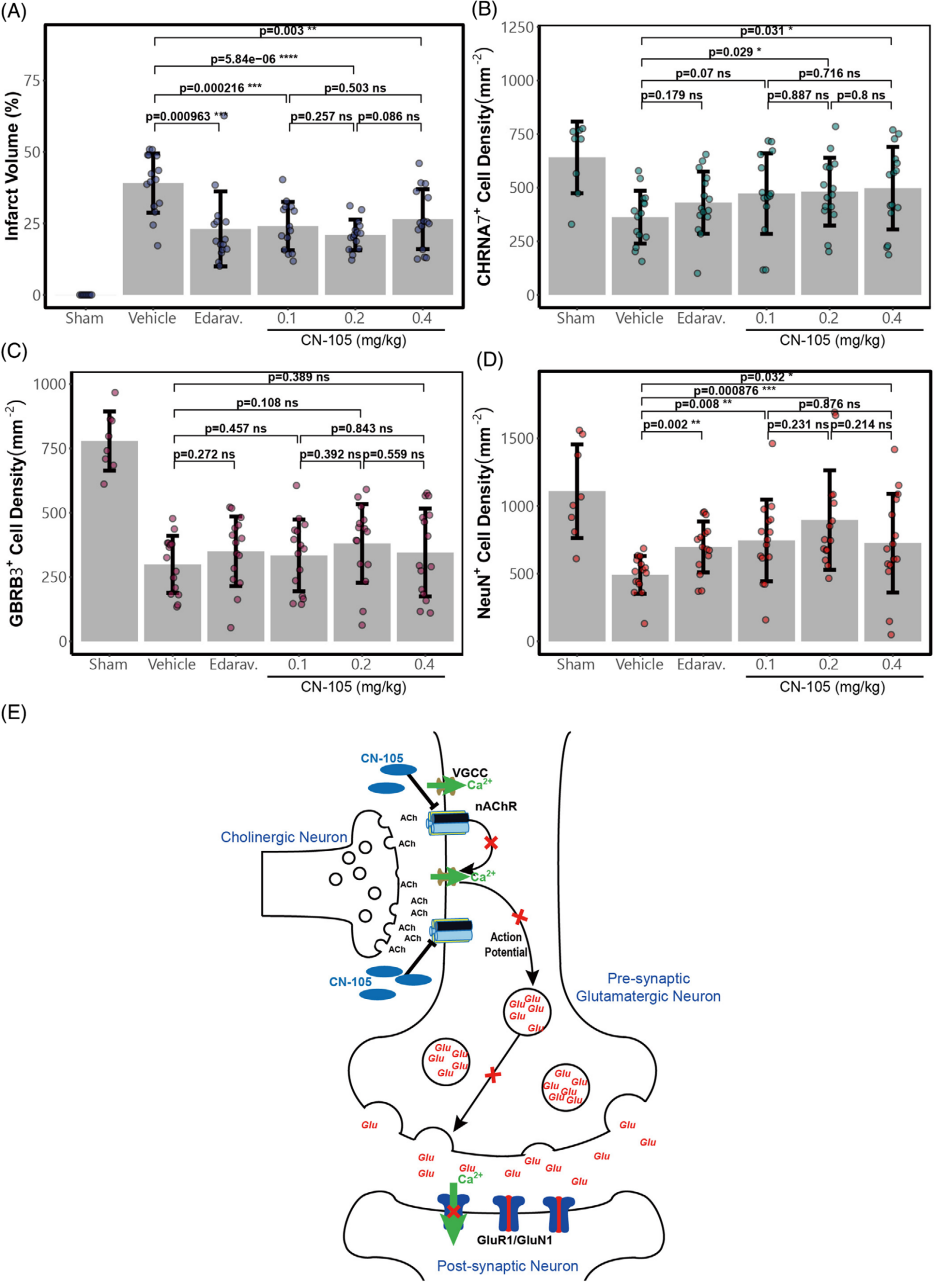
(A) Ratios of infarct volume over the total volume of ipsilateral hemisphere determined on Day 8 after the ischemia‐reperfusion injury. (B) The density of CHRNA7^+^ cells in a 1 mm^2^ sample area located on the margin of infarct determined on Day 8. (C) Density of GBRB3^+^ cells in a 1 mm^2^ sample area located on the margin of infarct determined on Day 8. (D) Density of NeuN^+^ cells in a 1 mm^2^ sample area located on the margin of infarct determined on Day 8. Statistics (*t*‐test) were calculated using the “rstatix” and “ggpubr” packages in R. (E) Mechanism of action for CN‐105's presynaptic suppression of glutamate release. For glutamatergic neurons receiving cholinergic signals, CN‐105's antagonism of nAChR reduced ACh‐induced action potential and calcium influx, limiting the glutamate release at its presynaptic terminal. Attenuated glutamate release led to reduced postsynaptic neurotransmission of excitatory signals

In conclusion, we demonstrated that one of the neuroprotective mechanisms of CN‐105 was the dampening of presynaptic glutamate release via nAChR inhibition, arising from a unique electrostatic gating effect on the cation channel. Our current observations emphasize that, in addition to its effects on glia, the direct interaction between apoE (and the peptide derivatives) and neuronal ion channels may play an important role in mediating its neuroprotective effects. Our results also suggest that it may be worthwhile to reconsider the role of nAChR as a potential therapeutic target for stroke neuroprotection.

## CONFLICT OF INTEREST

GC is an employee of Cerebtron Biotech. LY is an officer of ICE Biosciences, a contract research organization for conducting part of the electrophysiological assays. DTL is an officer and has equity in Aegis‐CN. Duke University has equity and a global intellectual property stake in CN‐105 and might benefit if proven effective and successful commercially. Cerebtron Biotech has equity and Chinese intellectual property stake in CN‐105. Cerebtron provided the study drug and part of the research funding. However, neither Cerebtron Biotech, nor Aegis‐CN had any editorial control over the study design, its execution, or the writing of this manuscript. Other authors declare that they have no competing interests.

## Supporting information

Supporting information
Materials and Methods
Figure S1 Pharmacokinetic curves of CN‐105 in Cyn monkey
Figure S2 ACh‐induced currents of α7‐nAChR in the presence or absence of CN‐105 (10 μM) or apoE3‐(1‐191) (5 μM)
Figure S3 (A) Structure models of CN‐105 bound to the α7‐α7 interface. The ribbon mode depiction of complex structures was generated in VMD and only the subunits involved in ligand binding were included for clarity. The snapshot structure of the simulation trajectory with the lowest interaction energy (calculated with the namdEnergy plug‐in of VMD) was used for the analyses. (B) The interaction diagram was generated with LigPlot+. Names of receptor residues involved in ligand binding were in green or black, while residue names of CN‐105 were in blue.
Figure S4 Amperograms of CN‐105 (200 nM) on AMPA‐mediated and NMDA‐mediated eEPSC in ex vivo rat brain slices. For each receptor, data from 8 independent recordings were collected.
Figure S5 Amperograms of CN‐105’s effect on GluR (AMPA‐R) in primary rat hippocampal neuronal cultures (Left) and on GluN (NMDA‐R) in a culture of HEK293 cells stably expressing NMDA receptor (Right)
Table S1 Pharmacokinetic parameters of CN‐105 in SD rat and Cyn monkey
Table S2 Inhibitory activities of CN‐105 homologues (200 nM) for α7‐nAChR relative to currents induced by 9 μM ACh (CN‐105 = Ac‐VSRRR‐NH2)

Supporting information.
Movie S1 Recording of C57BL6 mice undergoing CN‐105 injection (40 mg/kg) with mechanical ventilation.

## References

[1] James ML , Troy J , Nowacki N , et al. CN‐105 in participants with acute supratentorial intracerebral hemorrhage (CATCH) trial. Neurocrit Care. 2021. 10.1007/s12028-021-01287-0 34424490

[2] Wang H , Faw TD , Lin Y , et al. Neuroprotective pentapeptide, CN‐105, improves outcomes in translational models of intracerebral hemorrhage. Neurocrit Care. 2021; 35(2):441‐450. 33474632 10.1007/s12028-020-01184-y

[3] Gay EA , Klein RC , Yakel JL . Apolipoprotein e‐derived peptides block alpha 7 neuronal nicotinic acetylcholine receptors expressed in Xenopus oocytes. J Pharmacol Exp Ther. 2006;316(2):835‐842. 16249370 10.1124/jpet.105.095505

[4] Meloni BP , Mastaglia FL , Knuckey NW . Cationic arginine‐rich peptides (CARPs): a novel class of neuroprotective agents with a multimodal mechanism of action. Front Neurol. 2020;11:108. 32158425 10.3389/fneur.2020.00108 PMC7052017

[5] Albuquerque EX , Pereira EF , Alkondon M , Rogers SW . Mammalian nicotinic acetylcholine receptors: from structure to function. Physiol Rev. 2009;89(1):73‐120. 19126755 10.1152/physrev.00015.2008 PMC2713585

[6] Picciotto MR , Higley MJ , Mineur YS . Acetylcholine as a neuromodulator: cholinergic signaling shapes nervous system function and behavior. Neuron. 2012;76(1):116‐129. 23040810 10.1016/j.neuron.2012.08.036 PMC3466476

[7] Campbell BCV , De Silva DA , Macleod MR , et al. Ischaemic stroke. Nat Rev Dis Primers. 2019;5:70. 31601801 10.1038/s41572-019-0118-8

[8] Xi GH , Keep RF , Hoff JT . Mechanisms of brain injury after intracerebral haemorrhage. Lancet Neurol. 2006;5(1):53‐63. 16361023 10.1016/S1474-4422(05)70283-0

[9] Taly A , Corringer PJ , Guedin D , Lestage P , Changeux JP . Nicotinic receptors: allosteric transitions and therapeutic targets in the nervous system. Nat Rev Drug Discov. 2009;8(9):733‐750. 19721446 10.1038/nrd2927

[10] Gay EA , Bienstock RJ , Lamb PW , Yakel JL . Structural determinates for apolipoprotein E‐derived peptide interaction with the alpha 7 nicotinic acetylcholine receptor. Mol Pharmacol. 2007;72(4):838‐849. 17609418 10.1124/mol.107.035527 PMC2742887

