# Supplementary material for: Antagonism of nicotinic acetycholinergic receptors by CN‐105, an apoE‐mimetic peptide reduces stroke‐induced excitotoxicity
Source: Clin Transl Med. 2022 Jan 24;12(1):e677. doi: 10.1002/ctm2.677 (PMC8787096; doi:10.1002/ctm2.677)
Supplement: Supplementary file 1 — Supporting information Materials and Methods Figure S1 Pharmacokinetic curves of CN‐105 in Cyn monkey Figure S2 ACh‐induced currents of α7‐nAChR in the presence or absence of CN‐105 (10 μM) or apoE3‐(1‐191) (5 μM) Figure S3 (A) Structure models of CN‐105 bound to the α7‐α7 interface. The ribbon mode depiction of complex structures was generated in VMD and only the subunits involved in ligand binding were included for clarity. The snapshot structure of the simulation trajectory with the lowest interaction energy (calculated with the namdEnergy plug‐in of VMD) was used for the analyses. (B) The interaction diagram was generated with LigPlot+. Names of receptor residues involved in ligand binding were in green or black, while residue names of CN‐105 were in blue. Figure S4 Amperograms of CN‐105 (200 nM) on AMPA‐mediated and NMDA‐mediated eEPSC in ex vivo rat brain slices. For each receptor, data from 8 independent recordings were collected. Figure S5 Amperograms of CN‐105’s effect on GluR (AMPA‐R) in primary rat hippocampal neuronal cultures (Left) and on GluN (NMDA‐R) in a culture of HEK293 cells stably expressing NMDA receptor (Right) Table S1 Pharmacokinetic parameters of CN‐105 in SD rat and Cyn monkey Table S2 Inhibitory activities of CN‐105 homologues (200 nM) for α7‐nAChR relative to currents induced by 9 μM ACh (CN‐105 = Ac‐VSRRR‐NH2) [file CTM2-12-e677-s001.docx]

**Supporting information**

**Materials and Methods**

**Study Design**

In all animal assays of this study, animals were randomly assigned to each experimental group, to which the experimenters were blinded. Detailed grouping information was provided in the following sections. All *in vitro* experiments were performed in triplicates unless otherwise stated.

**Cells and animals**

Transiently transfected HEK293 cells: HEK293 cell line transiently expressing nicotinic ACh (α4β2) receptors was used. The patch clamp recording was performed on the third or fourth day after transfection based on the expression level of co-transfected GFP gene. Detailed description of the transfection procedures can be found in the Supporting information.

Stably transfected HEK293 cell line: HEK293 cells stably expressing nAChR α7/RIC3, were proprietary cell lines of ICE Bioscience. In a 24-well plate, 8×10^3^ cells were seeded on poly-D-lysine-coated coverslips and were allowed to grow for 18 h.

Rat primary neuronal culture: Primary neuron cultures were prepared from newborn rats. A mixed culture of hippocampal, cortical and thalamic neurons was used for calcium imaging assays. For patch clamp studies, hippocampal neurons from the CA1 region were extracted. The trypsinized brain tissue was incubated at 37°C for 20 minutes before the removal of the enzyme. The cells were washed three times with PBS and suspended with fresh media before being plated on poly-D-lysine-coated coverslips. After 4 h, the media was replaced by Neurobasal+B27+L-Glu (Thermo scientific, USA). Half of the culture medium was changed after 24 h before switched to a 72-hr medium change routine.

Oocytes: Mature oocytes of stage V and VI with smooth cell surface and no residual fibrous tissue and capillaries were selected. The cells, in a petri dish containing ND96 solution and Ca^2+^, were incubated at 17°C for later transfection of the nAChR α, β, δ and ε genes (Supporting information).

**Transfection and culture of cells for single cell electrophysiological studies**

**HEK293(Transient transfection)**: The HEK293 cells were cultured in a 6-well plate at a density of 5×10^5^ cells/well. The ratio of plasmid to transfection reagent was 1 μg: 2 μl. The total amount of plasmids per well was 3 μg. The Lipofectamine 3000 (Invitrogen) transfection reagent was used on day 2. The transfection medium was changed after 4-6 hrs. On day 3 the cells were digested and seeded on poly-D-lysine-coated coverslips placed in a 24-well plate at a density of 8×10^3^ cells per well.

**nAChR α4β2:** HEK293 cells were seeded on poly-D-lysine-coated coverslips placed in a 24-well plate at a density of 8×10^3^ cells per well. Cells were transfected with nAChR α4/nAChR β2/GFP with the ratio 2:2:1. The X-tremeGENE HP DNA (Roche) was used for transfection, and the ratio of plasmid to transfection reagent was 1 μg: 2 μL.

**HEK293 (stable expression)**: Cells stably expressing nAChR α7/RIC3 receptors were cultured in DMEM medium supplemented with 10% FBS, 800 µg/mL G418 and 200 µg/mL Hygromycin B; Cells were incubated at 37℃ with 5% carbon dioxide and humidity. Before manual patch clamp test, the cells were detached using 0.25%-Trypsin-EDTA solution. Then 8×10^3^ cells were seeded on poly-D-lysine-coated coverslips placed in a 24-well plate (final medium volume: 500 μL) and tested after 18 hours.

**Oocytes**: To investigate CN-105’s effect on the α1β1δε-nAChR receptor, borosilicate glass pipette (BF150-86-10，Sutter Instruments) was used to make the micro injector by the puller (P97，Sutter Instruments), then a section of the tip was cut off with tweezers and was filled with the mineral oil to make customized micro-injector. The four subunits of nAChR (α, β, δ and ε) at a concentration of 1000 ng/µL in a volume ratio of 1:1:1:1 were mixed in the RNAse free ultrapure water. The Micro-injector absorbed the cRNA (< 5 µL). For micro injection the micro electrode was advanced through the oocyte membrane until the needle tip passed through the cell membrane. Then, 35 nL of cRNA solution was injected into single oocyte. The injected oocytes were placed in ND-96 culture medium and incubated at 17°C for 48 hours (the medium was changed every day).

**GLP acute toxicology study**

The acute toxicology study of CN-105 was carried out at the facility of CTI Biotechnology (China) in accordance with GLP guidelines of the National Medical Product Administration (NMPA) of China. Briefly, six Cynologous (Cyn) monkeys were assigned to three dosing groups (n = 2/group, M:F= 1:1). The animals were given an iv. infusion of saline (Group 1), 25 mg/kg CN-105 (Group 2) or 100 mg/kg CN-105 (Group 3) twice a day. The infusion volume was 20mL/kg, while the infusion rate was 30min. Forty SD rats were assigned to four dosing groups (n = 10/group, M:F= 1:1) and were given an iv. injection of saline (Group 1), 15 mg/kg CN-105 (Group 2), 30 mg/kg CN-105 (Group 3) or 45 mg/kg CN-105 (Group 4) twice a day. The infusion volume was 5mL/kg, while the infusion rate was 10min. Animals were monitored for a variety of behavioral, physiological, histological and serological endpoints.

**Pharmacokinetics (PK) study of CN-105 in SD rat and Cyn monkey**

**Dosing of SD rats (PK):** Forty SD rats were randomly divided into four groups with both sexes. A single intravenous dose of 0.1 mg/kg, 0.3 mg/kg, 1 mg/kg or 3 mg/kg CN-105 was administered to rats. The infusion volume was 5mL/kg and given in 2min. Blood samples (300μL) from the jugular vein cannula were collected into EDTA-K_2_ tubes before and 2 min, 5 min, 15 min, 30 min, 1 h, 1.5 h, 2 h, 4 h, and 8 h after the administration of each dose. The blood samples were centrifuged at 1500 × g for 10 min at 4°C. The plasma treated with 1% HALT protease inhibitor was stored at -80°C until liquid chromatography-tandem mass spectrometry (LC-MS/MS) analysis for drug concentration.

**Dosing of Cyn monkeys (PK):** Twenty-four Cyn monkeys were used in this study. Cyn monkeys were randomly divided into three groups with both sexes, eight in each group. Animals in each group were individually administered an intravenous dose of 0.1 mg/kg, 0.5 mg/kg, 2.5 mg/kg CN-105. The infusion volume was 20mL/kg. A single dose of CN-105 was administered slowly over a 30 min period. Blood samples (800 μL) from non-administration venous limbs of A-C group Cyn monkeys were collected into EDTA-K_2_ tubes before and 0 min, 35 min, 45 min, 1 h, 1.5 h, 2 h, 3 h, 4 h, 6 h and 10 h after administration of each dose. The blood samples were centrifuged at 1500 × g for 10 min at 4°C. The plasma treated with 1% HALT protease inhibitor was stored at -80°C until LC-MS/MS analysis for drug concentration.

**Calcium Imaging Assays**

Calcium flux imaging: The cell (from 24-hour old rat pup) suspension was then seeded at a 2.5x10^4^ cells/well density in a 96-well plate (Thermo Fisher, USA) pre-coated with Matrigel (BD Biosciences, USA), and . Calcium Fluo-4 dye (Thermo Fisher, USA) were loaded according to the manufacturer’s protocol. Briefly, 0, 2 nM, 200 nM, 20 uM and 1 mM CN-105 was added to the imaging buffer prior to the imaging experiment. The plate was then loaded into the Cytation 5 instrument for continuous fluorescence imaging with 0.5s interval. A 30s baseline was first recorded before 40 uL of ACh solution was injected into the well via the auto-injector, reaching a final concentration of 1mM. The recording continued for another 30s before the automatic data analysis process. The summed fluorescence intensity of the images were used as the signal and data from five wells were collected for each concentration of CN-105 tested.

**Brain slice preparation**

The experiments were carried out on cultured hippocampal slices of SD rats, weighing 180-200 g. The rats were anesthetized (urethane, ip.; 25%, 1 ml/100 g) and killed by decapitation. After opening the cranial cavity, the brain was removed within 1.5 minutes. The brain was then moved to 0-4°C in sucrose-artificial cerebrospinal fluid (ACSF) (234 mM Sucrose, 2.5 mM KCl, 1.25 mM NaH_2_PO_4_•2H_2_O, 25 mM NaHCO_3_, 25 mM D-Glucose, 0.5 mM CaCl_2_, 10 mM MgSO_4_; pH:7.2-7.4) saturated with a mixture of 95% O_2_ and 5% CO_2_. The brain was then transferred into a petri dish containing cold ACSF (125 mM NaCl, 2.5 mM KCl, 1.25 NaH_2_O_4_•2H_2_O, 25 mM NaHCO_3_, 10 mM D-Glucose, 2 mM CaCl_2_•2H_2_O, 1.5 mM MgSO_4_, through (95% O_2_ and 5% CO_2_) mixed gas saturation pH=7.2-7.4 ). Then the cerebellum and about half of the frontal lobe were removed and the two hemispheres were separated. After removing the brainstem and the midbrain, the remained tissue blocks contained the hippocampus attached to the cortex. In this process, the ACSF was saturated with a mixture of 95% O_2_ and 5% CO_2_. Then the tissue blocks were fixed in a vibratome (Leica, VT 1000 S, Germany) holder containing cold ACSF, the blade's height was adjusted, and the hippocampus was transversely cut with a vibrating slicer, at a thickness of 300 µM. Throughout the process, the slices were saturated with a mixture of 95% O_2_ and 5% CO_2_. And the brain slices were immediately incubated in the ACSF for 30-60 min at 22-23°C before individual slice was transferred to a recording chamber.

After a brain slice was transferred to the recording chamber, the pyramid neurons of CA1 were visualized with the help of microscope’s differential interference contrast (DIC) mode. Patch pipettes containing intracellular solution (mM, 140 K-gluconate, 2 MgCl_2_, 10 HEPES, 8 KCl, 2 Na_2_-ATP, 0.2 Na_2_-GTP ) with 4-6 MΩ resistance were pulled from 110 mm long borosilicate glass capillaries (GB 150F-8P, Sutter instrument, USA). After established whole-cell patch clamp mode recording, spontaneous excitatory postsynaptic currents (sEPSCs) were obtained at a holding potential of -70mV. To pharmacologically isolate AMPA receptors (AMPARs) mediated excitatory currents, the GABA_A_ receptor antagonist bicuculline (10 µM) and the NMDA receptor antagonist 2-amino-5-phosphonopentanoic acid (50 µM) was applied in the bath. Addition 0.5 µM tetrodotoxin (TTX) which blocks fast and transient inward Na+ -dependent action potential-related transmission was perfused when mEPSCs were observed. Synaptically evoked currents were elicited by applying square wave pulses of 100 µsec duration and variable intensity through a glass electrode filled in ACSF with resistance of 800 KΩ located the Schaffer collaterals. To record the evoked AMPAR and NMDA receptor (NMDAR) mediated EPSCs (excitatory post synaptic currents), a certain threshold of stimulus intensity, below which no response could be detected, was defined. Then the recording stimulus intensity was kept at 1.5-fold of this threshold. AMPAR mediated evoked currents were recorded in normal ACSF and by setting the holding potential to –70 mV in presence of 50 µM D-AP5 and 10 µM bicuculline. Under these conditions, the evoked currents are dominated by activity from AMPAR. The stimulus-to-peak latency of AMPAR mediated currents was in the range of 4-6 ms and this latency was used at all other recorded membrane potentials to determine AMPAR mediated EPSCs. The peak amplitude of AMPAR mediated EPSCs was averaged from ten-minute recordings of stimulus at a frequency of 0.033 Hz. To isolate NMDAR mediated EPSCs, 20 µM NBQX  (2,3-dioxo-6-nitro-7-sulfamoyl-benzo [f] quinoxaline, an AMPAR antagonist), 10 µM bicuculline and 10 µM strychnine were applied. Cells were recorded at holding potential of +50 mV. To avoid eliciting action potential at this positive potential, intracellular cesium and QX314 ( *N*-(2,6-Dimethylphenylcarbamoylmethyl)triethylammonium) were used to block potassium and sodium channels.

**Solution formula used electrophysiological studies**

| **Channel** | **Extracellular Solution (mM)** | **Intracellular Solution (mM)** |
| --- | --- | --- |
| **mEPSCs/ sEPSCs** | ACSF:125 NaCl, 2.5 KCl, 1.25 NaH_2_PO_4_•2H_2_O, 25 NaHCO_3_, 10 D-Glucose, 2 CaCl_2_•2H_2_O, 1.5 MgSO_4,_ 95% O_2_ and 5% CO_2_, saturation pH is 7.2-7.4. | 140 K-gluconate, 2 MgCl_2_, 10 HEPES, 8 KCl, 2 Na_2_-ATP, 0.2 Na_2_-GTP. pH=7.3 with KOH. |
| **AMPAR mediated EPSCs** | ACSF, 50µM D-AP5, 10µM bicuculline, 10µM strychnine | 140 K-gluconate, 2 MgCl_2_, 10 HEPES, 8 KCl, 2 Na_2_-ATP, 0.2 Na_2_-GTP. pH=7.3 with KOH. |
| **NMDAR mediated EPSCs** | ACSF, 20µM NBQX, 10µM bicuculline, 10µM strychnine | 135 Cesium methanesulfonate,10 NaCl, 2 MgCl_2_•6H_2_O, 10 HEPES, 10 EGTA,2 Na2-ATP,0.2 Na_2_-GTP and 4mM QX314. pH=7.3 with CsOH. |
| **nAChR Currents** | 140 mM NaCl, 3.5 mM KCl, 1 mM MgCl_2_•6H_2_O, 2 mM CaCl_2_•2H_2_O, 10 mM D-Glucose, 10 mM HEPES, 1.25 mM NaH_2_PO_4_•2H_2_O_,_ pH=7.4 with NaOH. | 50 mM CsCl, 10 mM NaCl, 10 mM HEPES, 60 mM CsF, 20 mM EGTA, pH=7.2 with CsOH. |

**Model of transient middle cerebral artery occlusion (t-MCAO)**

Eighty-three Sprague-Dawley (SD) rats were randomly divided into six groups with both sexes as follows: 1) sham group (n=8), 2) vehicle group (n=15), 3) 0.1 mg/kg CN105 (n=15), 4) 0.2 mg/kg CN105 (n=15), 5) 0.4 mg/kg CN-105 (n=15), 6) 6mg/kg edaravone (n=15). Ischemic insult was introduced based on pre-established protocol. Briefly, rats were placed supine under anesthesia, and then the left common carotid arteries were exposed under sterile conditions. A suture was gently inserted into the internal carotid artery through the external carotid artery until the origin of the middle cerebral artery was occluded. The suture thread was removed 90 min after the ischemia, allowing restoration of the blood supply. Animals in the sham group were exposed of the left common carotid artery under anesthesia without ischemic occlusion. A single dose of CN-105 or edaravone were administered intravenously (iv.) 30min after reperfusion. The injection volume was 5mL/kg, given in 2 min. Animals in each group were euthanized at the end of the experiment. Brains were fixed in 10% formalin. The 3 mm-thick coronal brain sections were scanned and imaged to measure the percentage of viable and infarction brain tissue to calculate the percentage of cerebral infarction volume.

**Figure S1.** Pharmacokinetic plots of CN-105 in SD rat and Cyn monkey.

| 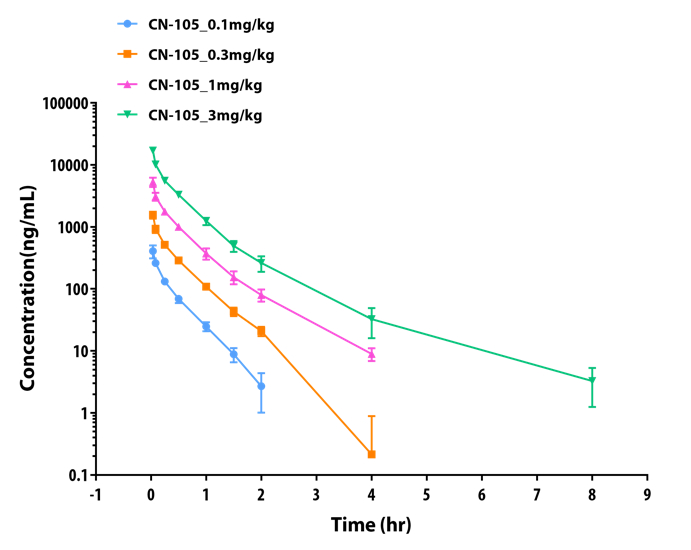 | 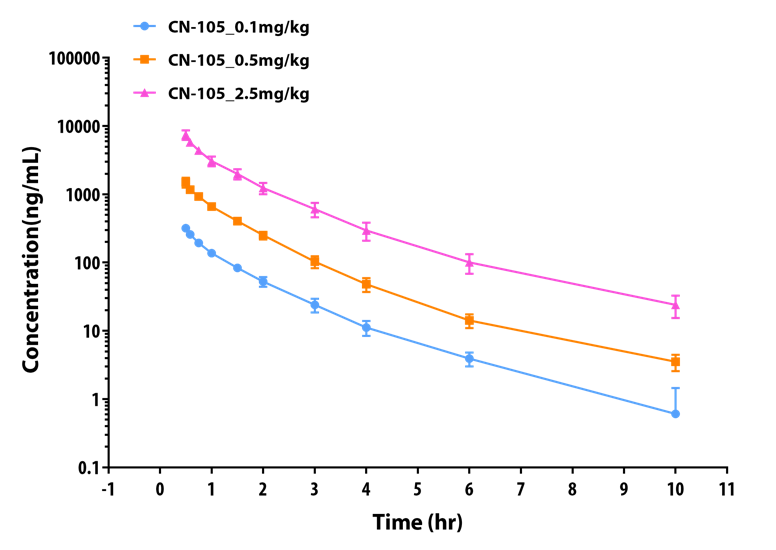 |
| --- | --- |
| 1. Concentration-time plot of CN-105 in SD rats after a single iv dosing at various dosages. | 1. Concentration-time plot of CN-105 in Cyn monkeys after a single iv dosing at various dosages. |
| 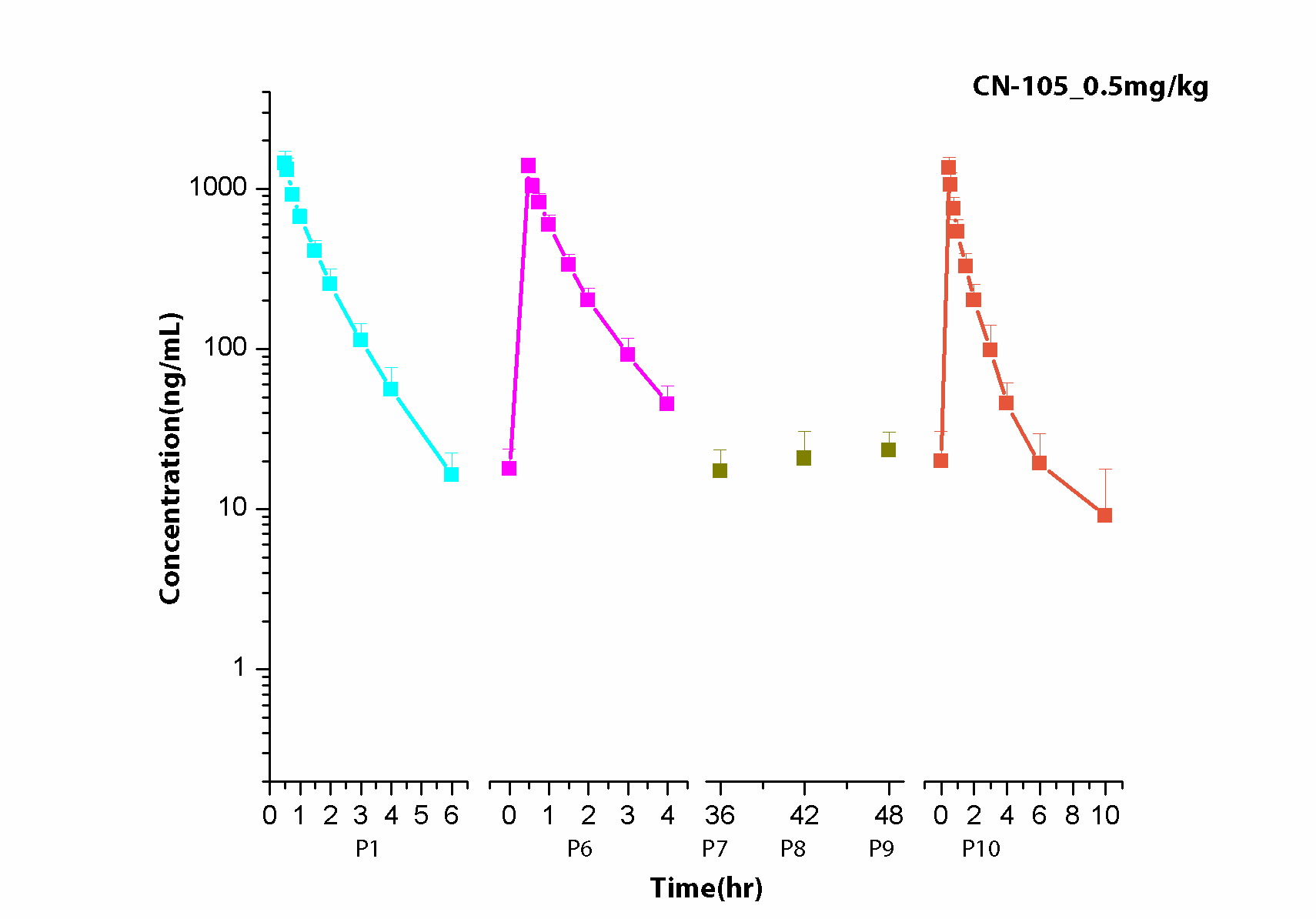 |  |
| 1. Concentration-time plot of CN-105 in Cyn monkeys with a multiple-dosing regimen at 0.5mg/kg. |  |
|  | |

**Table S1.** Pharmacokinetic parameters of CN-105 in SD rat and Cyn monkey.

| Does/  Parameters (Unit) | T_max_ (hr) | C_max_ (ng/mL) | C_0_ (ng/mL) | AUC_(0-t)_ (hr*ng/mL) | t_1/2_ (hr) | Kel (1/hr) | Vd (mL/kg) | CLs (mL/hr/kg) |
| --- | --- | --- | --- | --- | --- | --- | --- | --- |
| A (0.1 mg/kg) | 0.0383±0.0158 | 410±  90.5 | 560±  181 | 125±14.3 | 0.336±  0.042 | 2.09±  0.258 | 385±  71.1 | 793±85 |
| B (0.3 mg/kg) | 0.0333±0.0 000 | 1572±  211 | 2250± 463 | 504±56.6 | 0.405±  0.055 | 1.73±  0.203 | 342±  36.3 | 588±57 |
| C (1 mg/kg) | 0.0333±0.0  000 | 5315±  906 | 7679±  1735 | 1804±192 | 0.620±  0.0645 | 1.13±  0.106 | 500±  85.0 | 557±58 |
| D (3 mg/kg) | 0.0333±0.0  000 | 17050±1403 | 24048±  2357 | 5922±508 | 0.880±  0.170 | 0.823±0.204 | 645±  137 | 509±46 |

| Does/  Parameters (Unit) | T_max_ (hr) | C_max_ (ng/mL) | AUC_(0-10)_ (hr*ng/mL) | t1/2 (hr) | Kel (1/hr) | Vd (mL/kg) | CLs (mL/hr/kg) |
| --- | --- | --- | --- | --- | --- | --- | --- |
|  |  |  |  |  |  |  |  |
|  |  |  |  |  |  |  |  |
| A (0.1 mg/kg) | 0.5±  0.0 | 320±  20.2 | 348±33.7 | 1.38±  0.660 | 0.566±0.156 | 564±  271 | 285±28.4 |
| B (0.5 mg/kg) | 0.5±  0.0 | 1504±  251 | 1646±209 | 1.46±  0.171 | 0.481±0.058 | 642±  86.0 | 307±38.2 |
| C (2.5 mg/kg) | 0.5±  0.0 | 7460±  1192 | 8300±  1198 | 1.58±  0.200 | 0.445±0.054 | 691±  107 | 305±45.4 |

**Figure S2.** ACh-induced currents of α7-nAChR in the presence or absence of CN-105 (10 μM) or apoE3-(1-191) (5 μM).


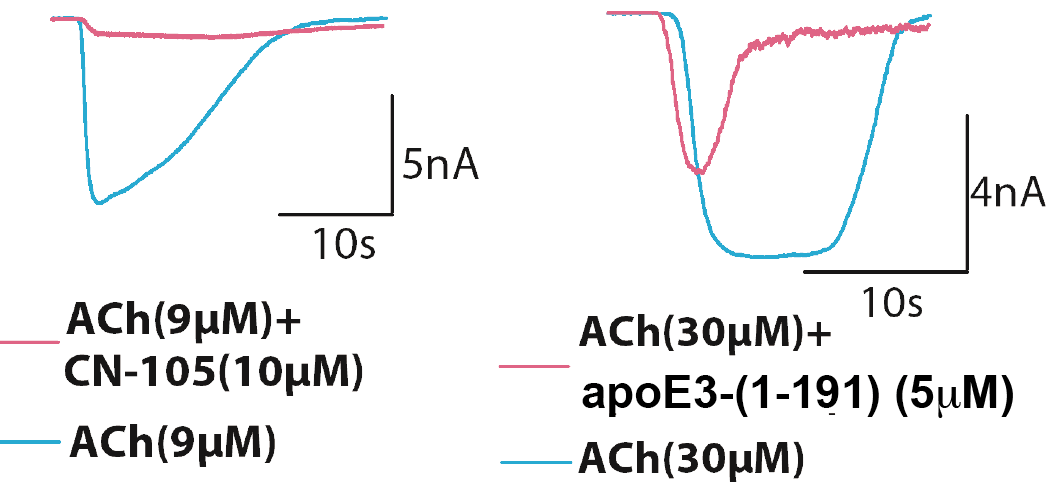


**Figure S3.** (A) Structure models of CN-105 bound to the α7-α7 interface. The ribbon mode depiction of complex structures was generated in VMD and only the subunits involved in ligand binding were included for clarity. The snapshot structure of the simulation trajectory with the lowest interaction energy (calculated with the namdEnergy plug-in of VMD) was used for the analyses. (B) The interaction diagram was generated with LigPlot+. Names of receptor residues involved in ligand binding were in green or black, while residue names of CN-105 were in blue.

| **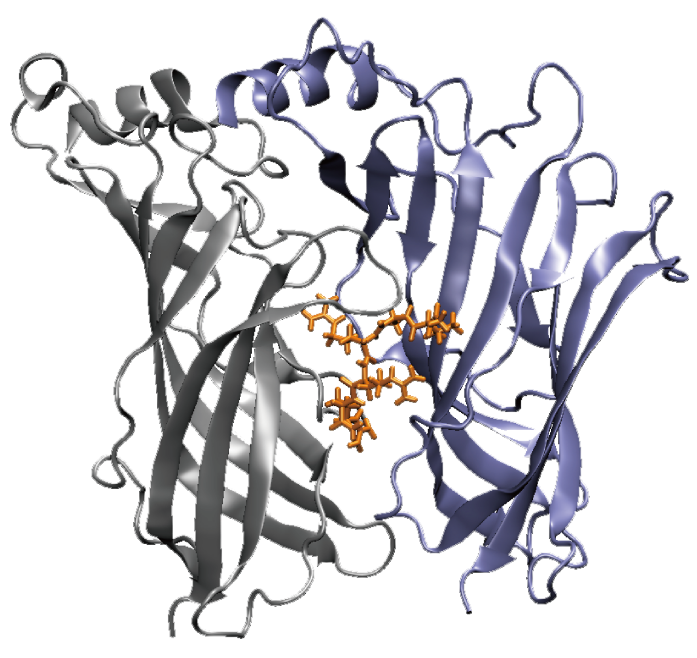** | **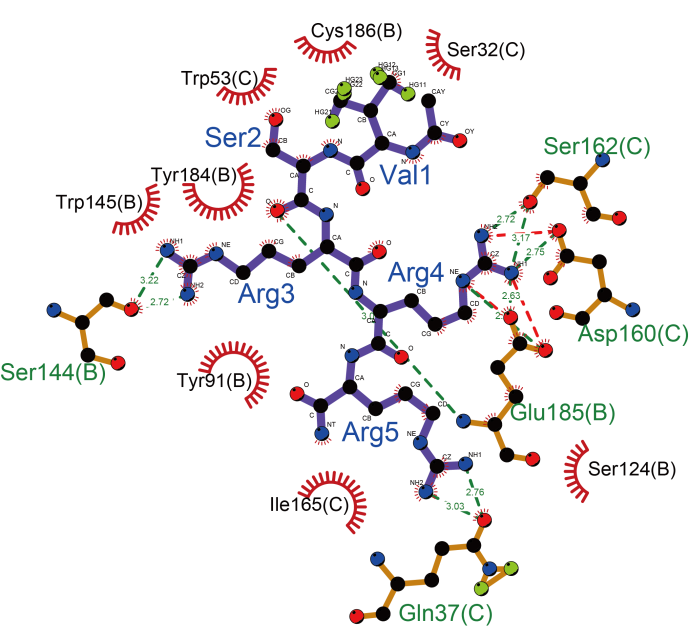** |
| --- | --- |
| (A) | (B) |

**Table S2.** Inhibitory activities of CN-105 homologues (200 nM) for α7-nAChR relative to currents induced by 9 μM ACh (CN-105=Ac-VSRRR-NH_2_).

| **Peptide** | **%Inhibition** | **Num of**  **+ charges** | **Difference from CN-105** | **Representative Amperograms** |
| --- | --- | --- | --- | --- |
| Ac-VSRRR-NH_2_ | 61.4% | 3 |  | |
| Ac-MSRRR-NH_2_ | 38.3% | 3 | V->M, large bulky residue | 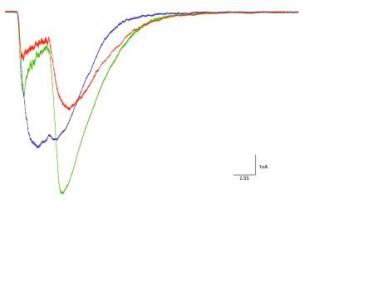 |
| Ac-ASRRR-NH_2_ | 7.4% | 3 | V->A, smaller side chain | 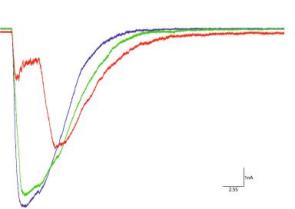 |
| Ac-RSKRR-NH_2_ | 9.3% | 4 | V->R, increase the charge | 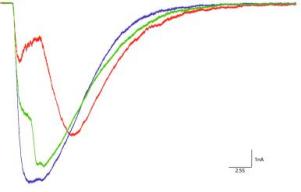 |
| Ac-VSKKR-NH_2_ | 48.9% | 3 | RR->KK, different residue | 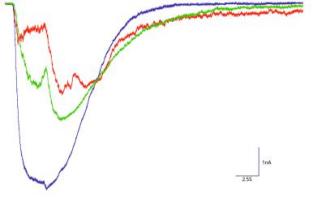 |
| Ac-VCRRR-NH_2_ | 5.9% | 3 | S->C, similar structure | 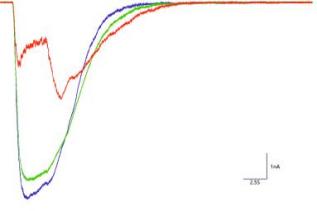 |
| TEELRVRLASHLRKLRKRLL | 75.2% | 5 | (ApoE130-149)  500nM | 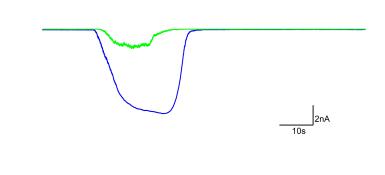 |

In the amperograms of Table S2, red traces were CN-105, blue traces were Control and green traces were from the peptide homologues (or ApoE130-149).

**Figure S4.** Amperograms of CN-105 (200 nM) on (a) AMPA-mediated and (b) NMDA-mediated eEPSC in *ex vivo* rat brain slices. For each receptor, data from 8 independent recordings were collected.

| 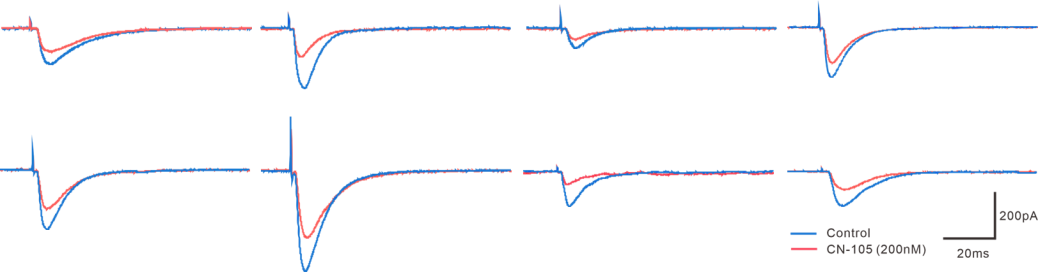 |
| --- |
| 1. AMPA-mediated eEPSC traces clamped at -70mV. (Blue: Control, Red: 200 nM CN-105) |
| 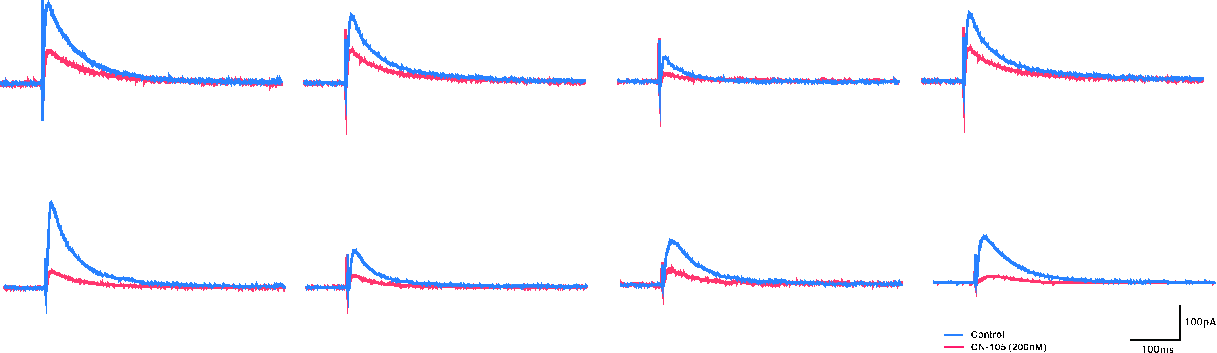 |
| 1. NMDA-mediated eEPSC traces clamped at +50mV. (Blue: Control, Red: 200 nM CN-105) |

**Figure S5.** Amperograms of CN-105's effect on GluR (AMPA-R) in primary rat hippocampal neuronal cultures (Left) and on GluN (NMDA-R) in a culture of HEK293 cells stably expressing NMDA receptor (Right).

| 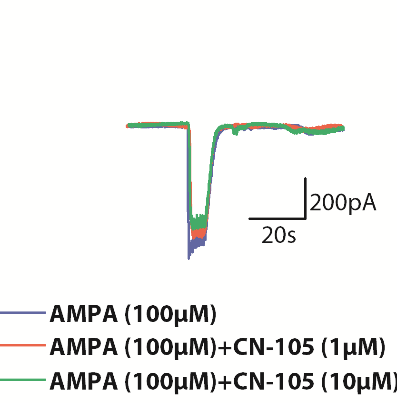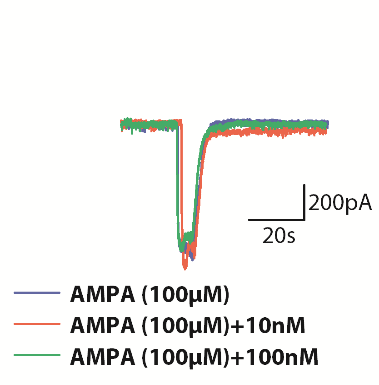 |
| --- |
| 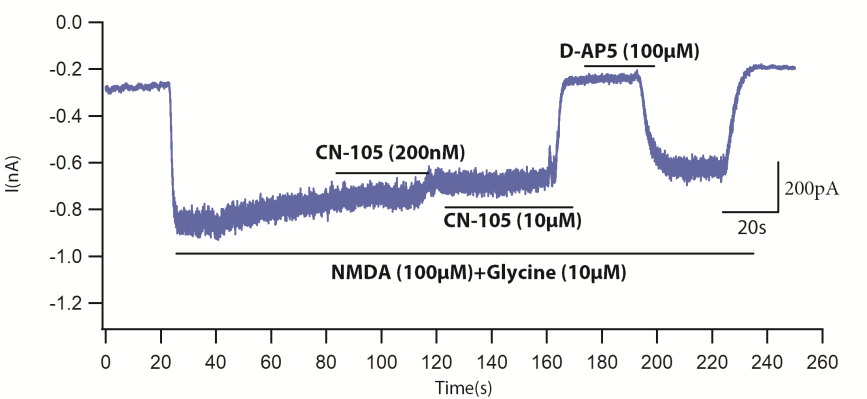 |

Movie S1. Recording of C57BL6 mice undergoing CN-105 injection (40 mg/kg) with and without mechanical ventilation.
